# Single-cell RNA sequencing in studies of type 1 diabetes mellitus: modern state-of-the-art and technical peculiarities

**DOI:** 10.3389/fendo.2025.1663728

**Published:** 2025-09-05

**Authors:** Azat Vadimovich Abdullatypov, Olga Valentinovna Glushkova, Ekaterina Sergeevna Petriaikina, Viktor Pavlovich Bogdanov, Dmitry Vyacheslavovich Tabakov, Vasilii Eduardovich Akimov, Vladimir Sergeevich Yudin, Anton Arturovich Keskinov, Sergey Mikhailovich Yudin, Pavel Yuryevich Volchkov, Dmitry Vladimirovich Svetlichnyy, Mary Woroncow, Veronika Igorevna Skvortsova

**Affiliations:** ^1^ Federal State Budgetary Institution Centre for Strategic Planning and Management of Biomedical Health Risks of the Federal Medical and Biological Agency (Centre for Strategic Planning, of the Federal Medical and Biological Agency), Moscow, Russia; ^2^ Institute of Basic Biological Problems, Russian Academy of Sciences, Federal Research Center “Pushchino Scientific Center for Biological Research of the Russian Academy of Sciences”, Pushchino, Russia; ^3^ Institute of Cell Biophysics, Russian Academy of Sciences, Federal Research Center “Pushchino Scientific Center for Biological Research of the Russian Academy of Sciences”, Pushchino, Russia; ^4^ Federal State Budgetary Scientific Institution “Federal Research Center for Innovator and Emerging Biomedical and Pharmaceutical Technologies”, Moscow, Russia; ^5^ Moscow Center for Advanced Studies, Moscow, Russia; ^6^ Moscow Clinical Scientific Center N.A. A.S. Loginov, Moscow, Russia; ^7^ Lomonosov Moscow State University, Moscow, Russia; ^8^ The Federal Medical Biological Agency (FMBA of Russia), Moscow, Russia

**Keywords:** type 1 diabetes mellitus, scRNA-seq, complete transcriptome analysis, cDNA libraries, third-generation sequencing

## Abstract

Type 1 diabetes mellitus (T1DM) is an autoimmune disease leading to destruction of pancreatic *β*-cells and loss of insulin production ability. Pathogenesis of T1DM is a complex process involving different types of immune cells, particularly, T-lymphocytes (including effector cells, Thelpers, regulatory lymphocytes, MAIT cells), B-lymphocytes, natural killers, monocytes, dendritic cells, and other minor cellular populations that form autoimmune response against islet cells. The heterogeneity of intercellular communications in the pancreas and adjacent lymph nodes of patients, as well as diverse functional state of islet cells, make a significant contribution to the pathogenesis of this disease. This makes the detailed consideration of immune cell subpopulations very essential for investigating the pathogenesis of the disease. Understanding the relations between changes of transcriptional activities in different cellular subtypes may allow to study the pathogenetic mechanism of T1DM in more detail, which could further be applied in both diagnostics and treatment. Single-cell RNA sequencing (scRNA-seq) allows to examine the interactions between immune cell subtypes and to identify differentially expressed genes specific for early stages of T1DM in particular cell subtypes. This review summarizes modern studies focusing on application of scRNA-seq for the studies of T1DM pathogenesis, novel biomarkers of manifestation, progression, and treatment efficiency for diabetes and its complications. The review covers studies on different cells and human tissues (endocrine, exocrine and immune pancreatic cells, PBMC) and model animals with experimental T1DM and its complications.

## 1 Introduction

Type 1 diabetes mellitus (T1DM) is an autoimmune disease accompanied by progressive destruction of *β*-cells in pancreatic islets, which leads to the loss of endogenous insulin secretion, hyperglycemia, and lifelong dependence of the patients on exogenous insulin administration. During the last decade, the overall number of people with this disease has been increasing (1). According to Type 1 Diabetes Index (URL: https://www.t1dindex.org/) citing the data published in The Lancet (1), by 2021, there were 630–000 patients with T1DM in China, 990–000 in India, 1 500–000 in the USA, 12–000 in Indonesia, 26–000 in Pakistan, 63–000 in Nigeria, 520–000 in Brazil, 27–000 in Bangladesh, 330–000 in Russia and 120–000 in Mexico. These numbers are strongly dependent on the quality of diagnostics in the healthcare systems of the countries - the prevalence varied from 1,5 per 100–000 in Papua New Guinea to 534 per 100–000 in Finland (2). According to the recent data (3), the list of the countries with the highest absolute number of patients with T1DM looks as follows: the USA - 1 477 000, India - 941 000, China - 599 000, Brazil - 499 000, UK - 341 000, Germany - 337 000, Russia - 323 000, Canada - 243 000, Saudi Arabia - 223 000, Turkey - 196 000. There were around 8.1 8.8 mln patients with T1DM in 2021 and 9.2 mln patients in 2025 all over the world (3). According to prognoses, by 2040 the number of patients with T1DM will reach 13.5-17.4 mln people (1).

The pathogenesis of this disease is not completely studied yet, and treatment methods are limited, resulting in the need for studies and innovations (4, 5). T1DM is known to be initiated by a wide range of factors in individuals with genetic predisposition. The clinical symptoms are caused by selective elimination of *β*-cells due to autoimmune attack mediated by CD4^+^ and CD8^+^ T-cells recruited by islet autoantigens; meanwhile, the presence of islet-specific autoantibodies is a prognostic risk factor for T1DM (6–9). As of today, around 90 genetic loci have been implicated in T1DM, containing risk factors that facilitate initiation and progression of T1DM; about a half of the known polymorphisms is situated in the region of human leukocyte antigen (HLA) genes, particularly, class II HLA, on 6p21 chromosome. Beside that, about 60 loci apart from HLA have been identified as reliably affecting the risk of the disease progression (10, 11).

**Figure 1:**
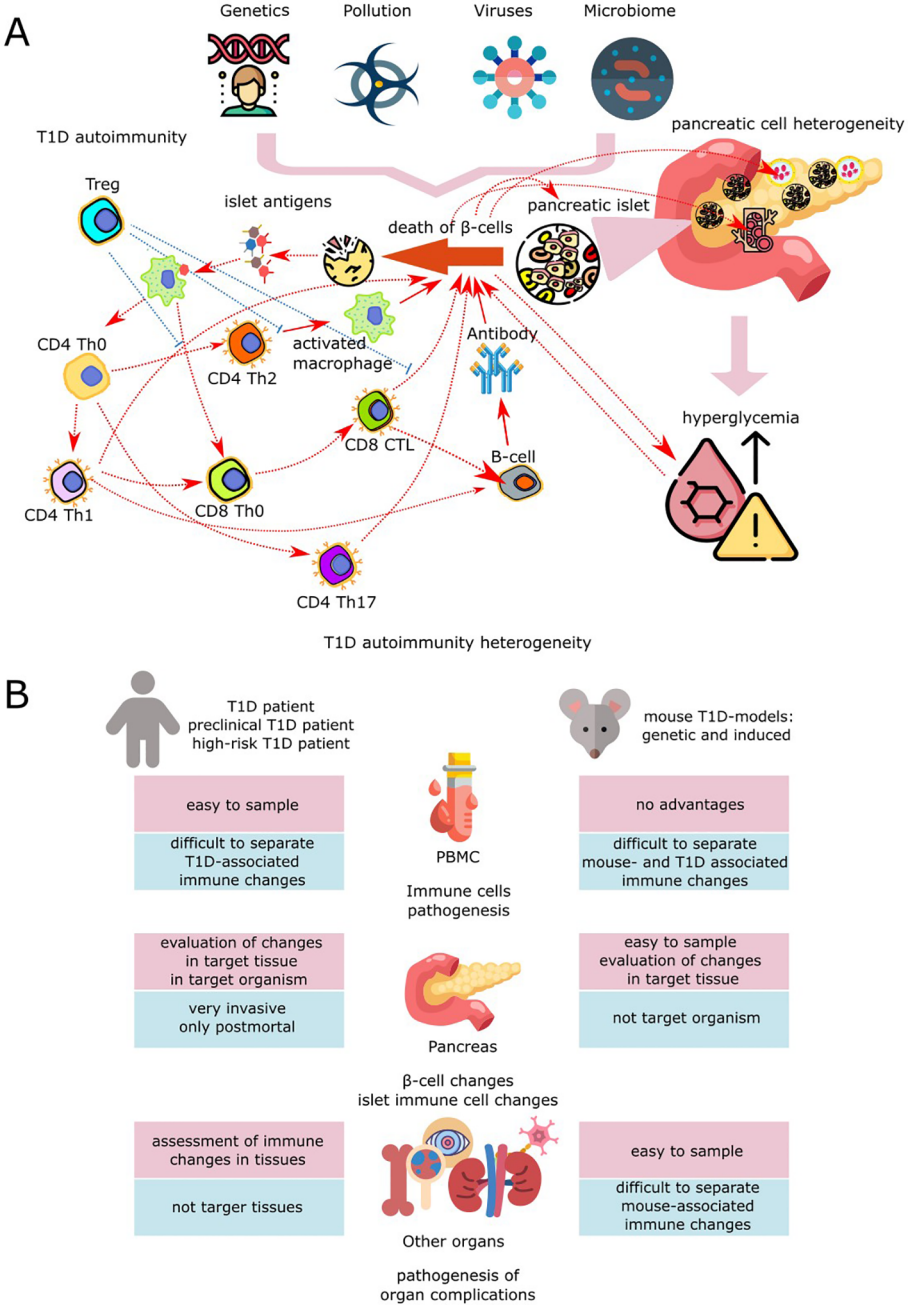
Application of scRNA-seq in biomedical studies of T1DM. **(A)** Pathogenesis of T1DM is determined by interaction between *β*-cells, other pancreatic cells and components of innate and adaptive immune systems. Initiation of the disease inpatients with genetic risk occurs upon the background of viral load, environmental pollution, pathogenic microbiome, and social stress (13). Among the viruses that are associated with an increased risk of progression of type 1 diabetes and activation of autoaggressive T cells, the most prominent are enteroviruses, particularly Coxsackie virus, echoviruses, as well as rotaviruses and, to a lesser extent, cytomegaloviruses, coronaviruses, viruses of mumps, parainfluenza, and rubella (14). Pathogenesis of T1DM is determined by interaction between *β*-cells, other pancreatic cells and components of innate and adaptive immune systems. Initiation of the disease in patients with genetic risk occurs upon the background of viral load, environmental pollution, pathogenic microbiome, and social stress (13). Among the viruses that are associated with an increased risk of progression of type 1 diabetes and activation of autoaggressive T cells, the most prominent are enteroviruses, particularly Coxsackie virus, echoviruses, as well as rotaviruses and, to a lesser extent, cytomegaloviruses, coronaviruses, viruses of mumps, parainfluenza, and rubella (14). Persistence of viruses in the pancreas leads to inflammation, causing *β*-cell stress with subsequent triggering of autoimmunity to islet antigens, virus-triggered cell death, immune-mediated loss of pancreatic *β*-cells and *β*-cell damage due to infection of surrounding cells. This contributes to the development of T1DM due to epitope spreading to islet neoantigens and critical antigens, molecular mimicry, bystander activation, addition of persistent infections, and virus-induced insulin resistance (15–18). Disruption of the composition and diversity of the intestinal microbiota initiates dysbiosis, promoting autoimmune activation due to excessive stimulation of receptors, chronic inflammation, barrier dysfunction, and immune dysregulation (19). Resident immune cells and peripheral blood cells, as well as islet, ductal, and epithelial cells of the pancreas, are involved in the formation of autoimmunity to pancreatic islet peptides. Initiation of the autoimmune process may occur as a result of the release of islet autoantigens due to the fragility and impaired proliferation and differentiation of *β*-cells, which demonstrate pronounced functional and adaptive heterogeneity in the pancreatic islets of healthy people (20). Changes in the functional activity and resistance to death of islet *β*-cells may also be associated with disturbances in their paracrine regulation by *α*-, *δ*-, *γ*/PP- and *ϵ*-cells of the islets, a decrease in exocrine-endocrine interactions with endothelial cells, as well as exocrine cells of the ducts and acini due to disruption of the functioning of each of these types of pancreatic cells. In T1D, innate and adaptive immune cells, including CD4^+^ and CD8^+^ T cells, B cells, and antigen-presenting cells (APCs), are involved in the dynamic progression of inflammation in the pancreatic islets of Langerhans, promoting the destruction and loss of *β* cells, which in turn leads to absolute insulin deficiency (7). Autoimmunity in T1DM occurs after infiltration of pancreatic islets by B cells and specific autoreactive T cells, which leads to the presentation of *β*-cell antigen peptides by antigen-presenting cells (dendrites and macrophages). The subsequent chain of activations of autoreactive CD4^+^ and CD8^+^ T cells contributes to the inflammatory destruction of *β*-cells and the recruitment of macrophages, natural killers, and neutrophils, which contribute to the progression of insulitis by releasing proinflammatory cytokines and reactive oxygen species. Defects in the functioning of tolerant Tregs and the stimulation of B cells by activated T cells in the pancreatic lymph nodes to produce autoantibodies to islet antigens lead to the complete destruction of insulin-producing cells (21). **(B)** Design of experiments used in modern biomedical studies for the examination of T1DM pathogenesis with scRNA-seq method.

In spite of a large number of studies, the pathogenesis of T1DM, including such aspects as primary triggers and exact mechanisms of islet autoimmunity, is still not clear. Theoretically, viral infections in early childhood leading to viral mimicry and expansion of T-cellular immunity to beta-cell antigens (12), as well as diet composition and gut microbiome dysregulation, can be the triggers of T1DM (**Figure 1A**). Other factors, such as toxins, chemical pollutants and stresses, can also be the potential initiators of this disease (10). On the other hand, damage, dysfunction and stress of *β*-cells and malfunction of their microenvironment in the islets could also be the primary trigger of autoimmune diabetes mellitus. One can suppose that in patients with high risk of T1DM these cells can already bear molecular signatures of the cellular stress and trigger autoimmune reactions due to accumulation of islet antigens upon their elevated death rate. The exact answer, whether the trigger of T1DM is the stress of beta-cells or impairment of immune cell tolerance, is yet to be given and interplay between these causes cannot be excluded as well (22).

Autoimmune processes that take place in T1DM development include different population of immune cells: CD4^+^ T-lymphocytes (naive and effector Th1, Th2, Th17, follicular T-helpers Tfh and regulatory CD4^+^ Treg) (23, 24), CD8^+^ T-cells (CD8^+^ stem T-cells, affected CD8^+^ T-cells, effector memory T-cells, central memory T-cells, exhausted CD8^+^ T-cells and regulatory CD8^+^ Treg) (25, 26), and B-cells (CD20^+^ B-cells, Breg, etc.) (27). Other immune cells, such as natural killers (28, 29) and monocytes (30) also affect the pathogenesis of T1DM. As all of these diverse cell populations involved in T1DM pathogenesis, most recent studies are focused on expanding the knowledge on the various cell signatures, signal pathways (31), and formation of intercellular interactions for all of these diverse populations that may be involved in T1DM pathogenesis (32). This direction seems especially important in the context of continuing search for effective treatment and early diagnostic biomarkers of T1DM. Single-cell RNA sequencing is a promising method for such studies, with its ability to scrutinize gene expression profiles on a single cell level in different tissues of T1DM patients, such as blood, pancreas, kidney, bone, bone marrow, etc. (**Figure 1B**). scRNA-seq reveals data on transcriptome of several thousands of cells from one sample, allows to classify them into cell types and draw comparisons between cell populations and their behaviors in different samples (33). Analysis of scRNA-seq data allows to reveal the relations between activity shifts in pancreatic and immune cells during T1DM progression, as well as determine the transcription alterations in other cell groups uncovering the mechanism of progression of diabetic complications (34).

The goals of the present review are to analyze the current scientific data on studies of pathogenesis of type 1 diabetes mellitus and its complications and to estimate findings in areas of early diagnostics and monitoring of treatment efficiency in T1DM patients by means of scRNA-seq.

## 2 scRNA-seq in biomedical studies

When compared with earlier methods of transcriptome analysis, such as microarrays and real time RT-PCR, RNA sequencing is more sensitive (compared to microarray) and much more informative as it captures wider sets of genes (35–37).

Sequencing the RNA of eukaryotic cells can be separated into two groups - bulk mRNA sequencing (bulk RNA-seq) and single-cell RNA sequencing (scRNA-seq), the latter being the topic of this review. The procedure of scRNA-seq consists of the following stages: 1) isolation of single cells from the cellular population; 2) isolation of RNA from each single cell and obtaining its cDNA copy; 3) preparation of libraries for sequencing; 4) sequencing of the libraries; 5) analysis of the obtained data. Several different protocols are used for the isolation of single cells. These protocols can be divided, regarding their technical basis, into the group of cytometric microfluidic protocols, where the single cells are collected by flow cytometry cell sorter or microfluidic device into separate wells (Fluidigm (38), BD Rhapsody (39), Singleron GEXSCOPE (40), and droplet (or emulsion) microfluidic methods, where a “water-in-oil” emulsion is created, in which each droplet of water contains a single cell and corresponding primer set immobilized on a bead (DropSeq, InDrop, 10x Chromium) (41–43).

scRNA-seq allows to scrutinize various physiological processes, such as maternal-fetal interaction (44), differentiation and functioning of complex organs (45, 46), aging and senescence (47), and to reveal minor subpopulations of immune cells that are invisible to other detection methods (48). This method is exploited in studying numerous immune processes, such as graft rejection (49), immune reaction in COLD (50), malignant gammopathies such as Waldenström macroglobulinemia (51), autoimmune diseases like systemic lupus erythematosus, rheumatoid arthritis, psoriasis (52–55). The common feature of these studies is the ability to determine certain cell subtypes that are most enriched in pathological processes and sets of genes expressed in these subtypes at high levels.

Data obtained by scRNA-seq are aggregated in specialized databases, usually in public access. One of the most useful databases on single-cell data for T1DM is HPAP (Human Pancreas Analysis Program). In 2023, the database contained data on 260,000 human pancreatic islet cells isolated from non-diabetic healthy donors (n = 31), AAb^+^ donors (with antibodies but without hyperglycemia) (n = 10), T1D patients (n = 9) and T2D patients (56).

## 3 Revealing the mechanisms of t1dm pathogenesis with scRNA-Seq

scRNA-seq demonstrated its applicability in studying various diabetic pathologies. It showed the possibility of influence of gestational diabetes on the fetus: the most specific feature was the elevated number of CXCL8^+^IL1B^+^ cells in PBMC from umbilical blood (57). Application of scRNA-seq to studies of type 2 diabetes mellitus (T2DM) revealed transcriptional shifts during immunometabolic changes in this pathology. PBMC analysis of T2DM patients revealed transcription changes in T-cells in patients with T2DM related to alteration of NF-*κ*B-, STAT3-, and FOXO1-dependent pathways (58, 59). Analysis of periodontitis or healthy tissues of patients with and without T2DM demonstrated complex expression patterns in macrophages possibly related to pathogenesis of this complication (60). Studying T2DM states in animal models with scRNA-seq has also brought useful insights. One such study revealed the key role of interferon 1 regulatory factor (IRF1) in progression of diabetic vasculopathy, a severe complication of T2DM (61), while another detected an essential decrease of Pdpn^+^ macrophage subpopulation capable of alleviating insulin resistance (62). Similarly, integration of scRNA-seq with bulk RNA-seq methods, ATAC-seq, and others, has also been a source of useful insights in T2DM, for understanding such complications as diabetic kidney disease or diabetic cardiomyopathy (63, 64).

Studying the pathogenetic mechanisms of T1DM by scRNA-seq method allows to determine the cellular subtypes participating at each stage of the diabetogenic process. Application of scRNA-seq also allowed to evaluate the molecular changes occurring in a single cell during the development and progression of the disease. This, in turn, made it possible to suggest some mechanisms of autoimmunity pathogenesis and the role of individual cellular subpopulations (**Figure 1A**) and pancreatic islet *β*-cells and their environment (**Figures 1A, B)** in manifestation and progression of T1DM. Studies are carried out using biological samples taken from patients with T1DM (peripheral blood - PBMC, rarer pancreatic tissue samples (65), and *in vitro* and *in vivo* models of this disease as well (**Figure 1B**). The subchapters below describe main cell groups participating in T1DM pathogenesis and the contributions made by scRNA-seq studies towards understanding their roles and functions in T1DM.

### 3.1 T-cells

Progression of autoimmune diabetes is related to the functional disbalance of effector and regulatory cells, and the disorder of immunoregulatory pathways can be the main factor determining the onset and progression of T1DM (66). Although almost all known populations of immune cells participate in T1DM pathogenesis, studies on human and animal cell culture models established a crucial role of T-cells in the development of islet autoimmunity and diabetes progression. Antigen-specific T-lymphocytes are the cells recognizing islet antigens and eliminating *β*-cells, thus participating in realization and regulation of autoimmune process (**Figure 1A**) (67, 68). Thus, studying the transcriptional alterations in the T-cells is often a focus in T1DM studies by scRNA-seq method.

A group of works is dedicated to studying the variants of antigen-recognizing cell surface receptors specific to T-lymphocytes of peripheral blood and pancreas (T-cell receptors, TCRs) and their affinity to the islet antigens determining the autoantigen-specific immune responses in T1DM (69). Profiling of TCR repertoire is a powerful tool for identifying the clones and phenotypes of T-cells directly involved in insulitis and degradation of *β*-cells. For example, Lai et al. (70) studied data from the GEO database for scRNA-seq on non-obese diabetic mice (NOD mice) and confirmed that the most prevalent cell type in the pancreas of diabetic mice were autoreactive CD8^+^ T-cells. TRAJ23 gene encoding the J-region of TCR alpha chain was upregulated in PBMC of T1DM mice according to scVDJ analysis, and its knockout alleviated the symptoms and severity of complications in mice with diabetes, while TRAJ-23 artificial T-cells had elevated toxicity to *β*-cells (70). Investigation of TCR clonotypes by scRNA-seq in parallel with full-transcriptome analysis of islet antigen-specific CD4^+^ memory T-cells of peripheral blood was carried out by Cerosaletti et al. (71). The cells were collected by a microfluidic device on a flow cytofluorimeter. RNA-seq of these cells revealed more extensive clonotype sharing among cells in T1D. Comparison of expanded and non-expanded T1DM T-cells’ expressions revealed upregulation of Th2-associated genes (GATA3, CCR4, and IRF4) and decreased expression of interferon response genes (IFNG, CD69, and GBP5). These results pointed at the heterogeneity of the functional state of autoreactive T-cells in patients with T1DM that involves activation of not just Th1 but also Th2 (71). Okamura et al. (72) studied TCR based on BD Rhapsody scRNA-seq of PBMC taken from T1DM patients and healthy subjects. They found:

- Higher expression levels of TCR alpha variables TRAV17 and TRAV21.
- Decreased diversity of CD8^+^ T cells and FOXP3^+^ regulatory T-cells in T1DM patients.
- CD8^+^ T-cells of T1DM patients showed elevated expression of PRF1, GZMH, ITGB2, NKG7, CTSW, and CST7.
- Decreased levels of CD4, CD7, CD5, HLA-A, CD27, and IL-32 transcripts.
- FOXP3^+^ T-cells showed enhanced expression of IL4R and TNFRSF4 in T1DM patients.

The authors suggested that the reduced variability of TCR in Tregs of T1DM patients may indicate their reduced immune tolerance (72). T-cells with affinity towards GAD65 (glutamate decarboxylase 65), an antigen present in pancreas and responsible for T1DM progression at early age, were studied by Eugster et al. (73) using single-cell RNA-seq in parallel with TCR sequencing. They found that although the amount of GAD65-specific T-cells was similar in patients and healthy donors, T1DM patients showed higher frequency of GAD-specific TCRB CDR3 sequences in the Treg subsets (73). Okamura et al. (74) used stimulation of peripheral blood by different pancreatic antigens (GAD65, IA-2 and insulin peptides) during cultivation of PBMC from blood samples of T1DM patients, followed by scRNA-seq analysis. They found that activation by insulin decreased the diversity of TCR repertoire compared to two other antigens and non-stimulated group. While there is a conventional view that autoimmune *β*-cell destruction is performed by CD4^+^ and CD8^+^ T-cells autospecific to islet antigens (75), recent studies on NOD mice revealed a high fraction (33%) of double negative (DN) T-cells in both peripheral blood and islet infiltrate, which could be a possible protective mechanism of peripheral tolerance (76).

Gearty et al. (77) revealed the mechanisms of effector pathways of *β*-cell-specific CD8^+^ T-cells in NOD mice during progression of T1DM by transcriptomic and clonal scRNA-seq analysis. They demonstrated that:

- *β*-cell-specific T-lymphocytes in peripheral lymph nodes are long-living stem progenitors.
- These can cause T1DM by differentiating and importing short-living autoreactive CD8^+^ mediator T-cells into the islets.
- Progenitor cells but not mediator cells are capable of inducing autoimmune diabetes.

Trivedi et al. (78) identified a subpopulation of stem-like CD8^+^ memory islet infiltrating T-cells with upregulation of:

- TIGIT (T-cell immunoreceptor with Ig and ITIM domains).
- PD1 (programmed cell death protein 1).
- EOMES transcription factor.

Their study revealed PD-1/PDL pathway in CD8^+^ T-cells as a key checkpoint of diabetic process, while TIGIT is located downstream of PD-1/PDL in this regulatory pathway.

Thus, T-cells are extensively studied by scRNA-seq in the context of their participation in autoimmune processes in T1DM, and the method allows to reveal fine differences between T-cell subpopulations that can hardly be detected by other methods.

### 3.2 B-cells

Beside T-cells, many other immune cell types are involved in the process of autoimmune destruction of pancreatic *β*-cells in T1DM. They perform both direct and mediating (regulatory) actions affecting the process of insulin-producing cell death (**Figure 1A**). Revelation of regulatory intercellular networks and determination of main populations and subpopulations of immune cells participating in T1DM is a subject of numerous scRNA-seq studies. The main subgroups of the mentioned immune cells are lymphoid (B-cells) and myeloid (monocytes and macrophages). Presence of autoantibodies in the blood of T1DM patients is a well-known fact. B-lymphocytes play a key role in the etiology of T1DM due to high expression of MHC (both class I and class II) and costimulatory molecules, autoantibodies and regulatory cytokines (79, 80). However, B-cells contribute to the disease not only as antibody producers but as antigen-presenting cells for T-cells allowing for the expansion of the pool of diabetogenic T-clones One of the functions of B-cells in manifestation and progression of T1DM is their help to the T-cells for expansion of the pool of diabetogenic T-clones. scRNAseq allowed to show transcriptomic changes of B-lymphocytes in autoimmune diabetes in both peripheral blood and pancreas. Altered expression of signaling pathway genes related to B-cell signal transduction and inflammation was shown in islet antigen-reactive B-cells (IAR B-cells) of autoantibody-positive donors and donors with T1DM. Particularly, these cells in PBMC samples from both T1DM group and Ab-positive donors had increased expression of genes related to antigen presentation (CD40, CIITA, and numerous HLA genes) compared to the control group (81). Using scRNA-seq, Luo and colleagues showed an increased number of naive B-cells with an upregulated coactivator of AKT-kinase, TCL1A, in humans and NOD-mice at early stages of T1DM. This gene was suggested as a diagnostic marker of T1DM risk and as a target for diabetes treatment by small interfering RNAs. Administration of antisense RNAs lowered the number of naive B-cells, rate of autoimmune *β*-cell degradation and led to better results of glucose intolerance test in mice with T1DM (82). Single-cell analysis of B cells in murine bone marrow after STZ induction of T1DM showed that the most upregulated genes were H2-Aa (encodes histocompatibility complex class II, antigen A, chain alpha), Ighd (immunoglobulin delta, heavy constant), Macf1(microtubule actin cross-linking factor 1), Ptpn22 (non-receptor protein tyrosine phosphatase, type 22), and H2-Eb1 (histocompatibility complex class II, antigen E, chain beta), while the most downregulated ones were Vpreb3 (pre-B-lymphocyte protein 3), Top2a(DNA topoisomerase II A), Mki67 (Antigen Kiel 67), Akap12 (A-kinase anchor protein), and Myb (myeloblastosis transcription factor) (83). However, none of these DEGs seem to be suitable as a candidate marker for T1DM due to the participation of all these genes in numerous processes and their ability to respond to various stimuli. Another study of B cells in the NOD mice diabetes model was aimed to clarify the relations between B-cell receptor affinity towards insulin and the phenotypes of B cells during T1DM progression. In this work, VH125 mouse model was used, a special line expressing the variant of immunoglobulin heavy chain, with two sublines - one with the possibility of T1DM progression (VH125.NOD) and another diabetes-resistant one (VH125.C57BL/6-H2g7). Insulin-reactive B-cells (IBCs) from NOD mice expressed higher amounts of IgM mRNAs and showed a tendency to increase in number with age and disease progression. While there was no significant difference between VH125.NOD and VH125.C57BL/6-H2g7 in the amount of low-affinity IBCs, the number of high-affinity IBCs was significantly higher in NOD mice (84). To summarize, scRNA-seq of B cells allowed to find at least one candidate for the role of T1DM biomarker (TCL1A), and further studies can reveal some new biomarkers and elucidate the mechanisms of B cell involvement into the pathogenesis of T1DM.

### 3.3 Other immune cells

There are several studies where scRNA-seq method was also applied for the determination of roles of circulating and resident antigen-presenting cells, the primary sensors of all the changes occurring in the islets. Zakharov et al. (85) analyzed transcriptomes of the following immune antigen-presenting cells in islets of NOD mice: conventional B-cells; plasma cells; classical dendritic cells (cDC); plasmacytoid dendritic cells (pDC); macrophages; stroma cells. The authors showed the heterogeneous character of the participation of these cell subtypes in the diabetogenic process: inflammation caused separation of cDC, and the greatest contribution was made by upregulation of immune-related and immune-activated genes Ifitm3, Cd40, Cxcl9, Ifitm2, Gbp4, Zbp1, Stat1, Isg15. scRNA-seq of macrophages of experimental and control animal groups revealed the basal inflammation-independent macrophage activation stage (stage 1), while the second stage was caused by inflammation (pseudotime 10–15 days in the experimental group of animals). The first stage was accompanied by elevated expression of Ccl3, Ccl4, Atf3, Cxcl13, the second one - by upregulation of pro-inflammatory genes Il18bp, Stat1, Il12b, Cxcl10, Psmb9, Cxcl8, Ccl5 Zakharov et al. (85) and Gao et al. (86) showed the role of monocytes in the pathogenesis of T1DM as a key subtype of immune cells with the greatest number of interactions with other cell subtypes. Monocyte activation was associated with differential expression of genes ACTG1, REL, TRIB1, their products being involved into signaling pathways PI3K/AKT/mTOR. High level of glucose *in vitro* stimulated activation of monocytes by elevating the expression of activation marker CD86 and proinflammatory cytokines IL-8 and TNF-*α*, accelerated monocyte apoptosis, changed the expression of the key genes (ACTG1, REL, TRIB1) and some genes of immune response (CXCL16, TGFBR1, CTLA4, CD48, TMIGD2, HLA-DPB1). Single-cell sequencing allowed to identify TRIB1 as a key gene for activation of monocytes and immune pathways upon hyperglycemic conditions (86. A comparison of single-cell transcription profiles of dendritic cells from patients with rheumatoid arthritis, systemic lupus erythematosus and T1DM showed specific signatures for each of the diseases. Dendritic cells from T1DM patients had downregulated levels of PTPN6 (non-receptor protein phosphatase type 6), TGFB (transcription growth factor beta), and TYROBP (tyrosine kinase binding protein) (87. Thus, application of scRNA-seq to transcriptomic studies in humans and animal models of pathogenesis of T1DM allowed to reveal the peculiarities of participation of different subpopulation of T-cells, B-cells, monocytes, macrophages and dendritic cells of peripheral blood and pancreatic islets in T1DM progression and helped to identify the role of TCR variants in autoimmune attack onto *β*-cells, details of autoreactive cell clonotypes, which is important to determine the pathogenetic significance of certain cell subtypes and variants. Revelation of novel subpopulations of immune cells and key genes of T1DM pathogenesis by scRNA-seq will promote development of personalized medicine and diagnostics of autoimmune diabetes.

### 3.4 Pancreatic cells

Human pancreas consists of endocrine part with the main function of regulating the glucose level in blood (88), and exocrine part secreting the enzymatic pancreatic juice. Endocrine part is comprised by islets of Langerhans, and the islet cells are divided into five types (main secreting hormones in parentheses): *α*-cells (glucagon), *β*-cells (insulin, C-peptide and amylin), *γ*-cells (pancreatic polypeptide), *δ*-cells (somatostatin) and *ϵ*-cells (ghrelin) (89). Functional secretion of these hormones is strictly regulated by metabolic and paracrine signals. Studies on the developmental biology of pancreas and heterogeneity of islet cells allowed to conclude that the pool of *β*-cells of both healthy and diabetic pancreas is very heterogenous, and this heterogeneity can be described quantitatively, taking into account the transcriptomic profiles of the islet cells. Direct signals from the surrounding tissues, including endothelial cells of blood vessels and neural cells, nutrients carried by blood vessels may affect the heterogeneity of maturation of endocrine cells. The subpopulations of epithelial cells were shown to have an important role in expression of insulin gene and *β*-cell proliferation (90). Due to the high invasiveness of the procedure of sample obtaining, the number of such studies is limited. For example, a review published in 2022 (91) mentioned only several T1DM studies by scRNA-seq (65, 92, 93). Nevertheless, scRNA-seq technologies are applied to study the differentiation of pancreatic islet cells and progression of their dysfunctional states related to T1DM. ScRNA-seq allowed to understand the transcriptional variety and functional heterogeneity of islet cells in normal and pathological conditions (90).

There is no doubt that the studies of proliferation, differentiation and functioning of the *β*-cells in the normal conditions and under autoimmune attack are of importance, given the context of T1DM. Integration of scRNA-seq and snRNA-seq (single nucleus mRNA sequencing) of human islet cells helped to reveal novel marker gene signatures of the islet cells beside the “conventional” genes used in clusterization of endocrine cell populations used in scRNA-seq analysis (INS, GCG, SST, and PPY for *β*-, *α*-, *δ*- and *γ*-cells, respectively). These markers are: ZNF385D, TRPM3, LRFN2, PLUT (for *β*-cells); PTPRT, FAP, PDK4, LOXL4 (for *α*-cells); LRFN5, ADARB2, ERBB4, KCNT2 (for *δ*-cells) and CACNA2D3, THSD7A, CNTNAP5, RBFOX3 (for *γ*-cells). These markers can serve as the indicators of proper differentiation of grafted islet cells (94). In the same scRNA-seq study, *β*-cells were divided into three subtypes. *β*1-cells are enriched by mature mRNA of insulin gene, they are actively producing insulin, but have low ability to proliferate; *β*2-cells are intermediate, and *β*3-cells are enriched by immature insulin gene mRNA, they have high proliferation ability but low secretion of insulin.

Key genes responsible for proliferation and maturation of mature insulin-producing cells were determined by scRNA-seq: SPOCK2 gene encoding an extracellular calcium-binding proteoglycan (without this protein, the human *β*-cells had elevated expression of MMP2 protease and activated *β*-integrin-FAK-c-JUN pathway, which led to significant acceleration of apoptosis). Treatment by MMP2 led to elevation of glucoseinduced insulin secretion by *β*-cells (95). Single-cell sequencing also determined SIRT2 as a suppressor of *β*-cell proliferation in hyperglycemic conditions. Inactivation of Sirt2 affected the transcriptional response of mouse *β*-cells to hyperglycemia and downregulated the hyperglycemia-activated signatures UPR (“Unfolded Protein Response” - misfolding stress proteins Herpud1, Hspa5, and Wfs1) and genes of ribosomal proteins (Rplp0, Rpl6, Rpl8, and Rps27a) (96).

Based on the modern paradigm, heterogeneity of functional activity of islet *β*-cells in T1DM depends on their microenvironment, particularly, on the state of endocrine and exocrine resident pancreatic cells, which is confirmed by novel findings describing the diabetogenic mechanisms (97–99). Modern view on T1DM emphasizes that alpha-cell dysfunction is also a symptom of T1DM (100–102). A recent study showed an interesting phenomenon: long-term intermittent fasting, a popular nutritional regime with several metabolic benefits, caused dysfunction of *β*-cells in adolescent mice, that was characterized by specific transcription pattern revealed by scRNA-seq. This pattern looked similar to that of humans with T1DM: not only the fractions of *α*- and *β*-cells were decreased in the overall cell population, but also the levels of some other transcripts related to diabetes progression (Mafa, Ins1, Ins2, Slc2a2, Nkx2-2, Pdx1, Pax6, Neurod1, Pdx6-1) were downregulated. So, the authors supposed that the altered food consumption regime in adolescence might be one of the causes of T1DM (103).

A discovery of PEG-modified insulin and GLP-1-estrogen conjugates as stimuli for *β*-cell redifferentiation at T1DM was also made using scRNA-seq: this method was used to track the expression of endocrine cell marker gene NGN3, and the therapies by these agents effectively reduced its level in delta-cells in STZ-induced diabetic mice (104). Application of scRNA-seq demonstrated that dysfunction of *α*-cells occurs before the major *β*-cell loss. Phenotypic classification of donors without T1DM but with elevated level of GADA autoantibodies (GADA+), showed that their GADA^+^ *α*-cells are characterized by changes in expression of glycolysis signaling, oxidative phosphorylation and cAMP signal transduction. Transcripts of PKIB, a cAMP-dependent protein kinase *β* inhibitor gene, were downregulated by on average 4.9 times in the *α*-cells from donors with high titer of GADA^+^ autoantibodies compared to healthy control donors, which means that the cAMP signaling pathway is activated in this group of donors (confirmed by elevated expression of CREB gene encoding cAMP-responsive element binding protein in GADA+). Glycolysis-gluconeogenesis pathway was downregulated, the gene of crucial glycolysis enzyme, aldolase, showing the maximal difference between GADA^+^ and control donors. As for oxidative phosphorylation, there were 15 significantly downregulated genes of this metabolic pathway in GADA^+^ (105).

Use of scRNA-seq allowed to find transcriptional changes of the cells of the exocrine part of the pancreas in T1DM. It was shown that in T1DM patients, the ductal cells express class II MHC and interferon at high levels. Transcriptionally, they are similar to the tolerogenic dendritic cells (65). Mesenchymal subpopulations enriched by factors related to TGF*β*, Hippo and inhibitor of DNA-binding (ID) signaling pathways were identified as necessary for endocrine differentiation of *β*-cells (90).

## 4 Searching for diagnostic markers of T1DM with scRNA-seq method

scRNA-seq allows to reveal genes that can further serve as diagnostic markers when detected by more simple methods like real-time PCR or microarrays. When compared to bulk RNA-seq, scRNA-seq has an advantage of using the two-coordinate system where the first coordinate is cell type, and the second coordinate is a set of differentially expressed genes. Hence, a number of marker phenotypes revealed in one sequencing study could increase dramatically. After finding the marker phenotype (cell type: DEGs), the diagnostics should comprise proper cell sorting and either real-time PCR or microarray analysis; another option could be the direct protein measurement or enzymatic activity measurement, but Frørup et al. (106) used publicly available datasets of scRNA-seq to ensure that the upregulated expression of cathepsin S gene (CTSS), a protease, is specific for *β*-cells in T1DM; the high level of CTSS expression in children was also shown to correlate with the presence of autoantibodies to the antigens of *β*-cells. Further examination revealed that *β*-cells secreted this protease under the action of pro-inflammatory cytokines. Higher level of this enzyme in children with the early stage of T1DM and all the aforementioned data allowed the authors of the study to conclude that this protease can serve as a potential marker of early T1DM in children (106).

The already mentioned paper of Gao and colleagues where three key T1DM-related genes were found, namely ACTG1, REL, TRIB1, showed that the coordinated expression of these genes in T1DM and in normal conditions allowed to presuppose their use as diagnostic markers; physiological relevance of their expression was also confirmed by *in vitro* experiment - the knockdown of TRIB1 in monocyte culture significantly decreased the activation of monocyte response by glucose (86).

Guo et al. (107) found a novel fraction of classical monocytes of peripheral blood (cluster 4) which had upregulated sialoadhesin gene expression, SIGLEC1. The portion of these monocytes was significantly higher in patients with latent autoimmune diabetes (LADA, subtype of T1DM). In murine T1DM model, the highest number of such monocytes in peripheral blood was found at 6 weeks of age, while in pancreatic tissues, their number reached maximum in 12 weeks. These monocytes also secreted a higher quantity of chemoattractants for T-cells and natural killers. The presence of monocytes with higher expression of sialoadhesin gene might further be used in clinical practice for earlier diagnostics of T1DM (107).

Another study used a combination of bulk RNA-seq and scRNA-seq for early detection of autoimmune diabetic states in children. The study used peripheral blood samples of children from 3 to 36 months of age of children with T1DM genetic predisposition. This work revealed a set of transcripts elevated earlier than T1DM-associated autoantibodies were detected. Among these transcripts, the most notable was IL32, mainly expressed by T-cells and NK-cells. Interestingly, expression of this interleukin in islets and particularly in *β*-cells can be stimulated by viral infection (Coxsackie virus CBV-1-7) and proinflammatory cytokines. The authors proposed IL32 as a candidate for further functional studies (108).

## 5 Estimation of treatment efficiency by scRNA-seq

Conservative treatment of type 1 diabetes mellitus presupposes lifelong insulin administration. Along with insulin therapy and replacement therapy, the most recent achievements in uncovering the mechanism of T1DM as well as recent progress in monoclonal antibody technology led to development of immunotherapy targeting the immune disorders leading to *β*-cell degradation.

Teplizumab, a monoclonal anti-CD3-antibody, is approved in a number of countries for immunotherapy of T1DM. scRNA-seq was applied to analyze transcriptome of the patients after 14-day teplizumab therapy courses. Teplizumab or placebo were administered to stage 2 T1DM patients at three time points: 0 (n=7), 3 months (n=7) and 18 months (n=7), and the clinical symptoms of T1DM (i.e. transition to stage 3) were tracked for 5 years. In 36% of cases, where the specific symptoms of T1DM did not manifest for 5 years, teplizumab administration caused notable transcriptional responses in CD8^+^ cells, such as decreased expression of interleukin receptor IL7R and another important marker, CD127 (109).

There are other antibodies that are suggested to be therapeutic agents for T1DM. (110, based on RNA sequencing of pooled cells, showed that an experimental antibody to TLR4 induced production of myeloid suppressor cells and thus suppressed the development of T1DM. In this work, not scRNA-seq sensu stricto was used, but total RNA sequencing of cellular subtypes that were sorted on a flow cytofluorimeter before; so, the work possessed functionality comparable to that of scRNA-seq (110). Based on scRNA-seq Lai and colleagues demonstrated that TRAJ23 could be a potential target for antibodies and supported their findings with knockout of its gene in mice leading to alleviation of T1DM symptoms in NOD mice model (70).

Balmas et al. (111) used scRNA-seq to search the biomarkers of response to alefacept, an engineered LFA-3/immunoglobulin fusion protein, at T1DM, and evaluated the reaction of anti-islet CD4^+^ T-cells to the therapy. The cells with diagnostically valuable phenotype (memory-like islet-antigen reactive CD4^+^ T-cells) formed a special cluster and their number had negative correlation with C-peptide content. Cpeptide persisted in about 30% of patients with the new-onset T1DM. Number of the novel phenotype of islet-reacting T-cells with elevated expression of BHLHE40 and genes of pro-inflammatory cytokines GM-CSF, TNF-*α*, IFN-*γ*, IL-17A, IL-2, is suggested by the authors as a marker of response to alefacept in T1DM patients (111).

Also noteworthy are the studies where scRNA-seq is used to determine the possibility of transplantation of artificially grown pancreatic islets for restoration of the lost endocrine functions. Pluripotent stem cells capable of differentiating into insulin-secreting *β*-cells and other endocrine cells are regarded as a source of islets for transplantation. However, the *in vitro* cultivated secretory *β*-cells are transcriptionally and functionally different from mature healthy native *β*-cells (112). During the study of transcriptional changes in the secretory *β*-cells after transplantation, scRNA-seq revealed an increase in similarity to native *β*-cells in transcription patterns. Insulin secretion and IAPP protein production increased, and the genes specific for the late maturation studies and inactive *in vitro* cultures were expressed, namely INS, MAFA, CHGB, and G6PC2. Other types of secretory cells, SC-*α* and SC-enterochromaffin cells (SC-EC), also displayed significant changes after transplantation (113).

Integration of scRNA-seq and snRNA-seq identified new gene signatures of *α*-cells (PTPRT, FAP, PDK4, LOXL4), *β*-cells (ZNF385D, TRPM3, LRFN2, PLUT), *δ*-cells (LRFN5, ADARB2, ERBB4, KCNT2) and *γ*-cells (CACNA2D3, THSD7A, CNTNAP5, RBFOX3), proposed for the role of markers of correct differentiation of the transplanted cells (94).

In a preprint describing enrichment of suspension of islets grown from stem cells by magnetic particles specific for the cell surface marker CD49a, scRNA-seq was used to confirm that the islets enriched with CD49a-positive cells had higher fraction of *β*-cells and more mature islet-like transcription patterns compared to the initial suspension (114). Also, scRNA-seq was applied to test the adequacy of an islet cryopreservation method (115) - in this work, the expression of genes responsible for synthesis of cell adhesion molecules EpCAM and HPi2, was shown to decrease right after thawing but to restore to the normal values in 24 hours, while other gene groups showed high expression both right after thawing and further. This meant that the method used for cryopreservation was appropriate, and the procedure did not cause any irreversible harm to the islets. scRNA-seq helped to estimate the efficacy of redifferentiation of *α*-cells and their reprogramming into *β*-cells from the point of view of specific marker gene expression (116), as well as the analogous process for pancreatic duct cells (117) - both these approaches can help to restore the *β*-cells during T1DM, since patients have enough *α*-cells and duct cells to subject them to reprogramming. The overall advantage of scRNA-seq compared to other expression analysis methods is its ability to sort those differentiated cells that are the most similar to mature *β*-cells in their transcription patterns, particularly, by the genes that determine secretory phenotype. Studies comparing allogeneic and syngeneic grafts of pancreatic islets allow to determine the signaling pathways involved into pancreatic graft rejection, for example, those regulated by pro-inflammatory cytokines (44, 118, 119). Combination of scRNA-seq with proteomics allowed to find the importance of phosphorylation of key proteins of *β*-cell differentiation, NKX6–1 and chromogranin A, which is also important for the development of *β*-cell transplantation method as a way to treat diabetes - proper level of these proteins might be used as a marker of correct differentiation (120). Another application of studying the dysfunction of *β*- and *α*-cells by scRNA-seq is control of dedifferentiation of *α*- and *β*-cells and reprogramming of islet cells as a method of T1DM therapy (121).

## 6 Examination of diabetic complications by scRNA-seq

scRNA-seq can also help to analyze molecular reasons and molecular patterns of complications related to diabetes.

Chronically decompensated carbohydrate metabolism is inevitably reflected on the condition of vascular walls and nerve fiber sheaths in patients with T1DM and T2DM. Diabetic foot ulcer, diabetic polyneuropathy, diabetic angiopathy and diabetic kidney disease are the most widespread examples of such late complications. Since complication risk management is important for maintaining the quality of life of the patients, methods for early recognition and prevention of such conditions exist and are developed in abundance. scRNA-seq method allows to detect specific markers of these late T1DM complications and to propose targets for their effective treatment. The best results were shown in T1DM studies where several methods were integrated.

Diabetic kidney disease is the most common complication of T1DM that occurs in 20 to 75 percent of all the patients. It was addressed by Wang et al. (122) who analyzed data from scRNA-seq and bulk RNA-seq, and these two methods gave 106 common DEGs at diabetic kidney disease. Generally, these genes were associated with the focal extracellular matrix adhesion pathway. The overlapping of this gene set with differentially expressed microRNAs gave a network of 60 mRNAs, 10 miRNAs and 5 non-coding RNAs. As a result, five key genes (VCAN, TIMP1, TNC, C3, and CP) differentially expressed in fibroblasts and renal tubular epithelium associated with glomerular filtration rate, creatinine level and proteinuria were found (122).

Lu et al. (123) analyzed three samples from patients with diabetic kidney disease and three samples of healthy donors for which the scRNA-seq data were available. The complication was found to correlate with upregulated genes of mTOR pathway in immune cells, and the key genes were translation initiation factor EIF4B, mTOR-complex subunit RICTOR and protein kinase C-beta PRKCB (123). Due to the changes of inflammatory status of the body, T1DM increases the risk of clear cell carcinoma of the kidney. One of the recent works compared scRNA-seq data of patients with these two diseases and revealed three key genes with altered expression in both these states, KIF21A (involved in microtubule dependent transport), PIGH (involved in glycosylphosphatidylinositol (GPI)-anchor biosynthesis and expression of proteins on the cell surface), and RPS6KA2 (controls cell growth and differentiation). The first two genes were downregulated compared to control, and they were mostly expressed in T-cells and NK-cells; the third gene was upregulated, and its transcripts were present in epithelial and endothelial kidney cells. On the whole, the expression of these genes might serve as a clear cell carcinoma risk marker in T1DM patients (101).

scRNA-seq proved the crucial role of macrophages in healing of chronic diabetic wounds. The analysis of CD45^+^ cells (monocytes, lymphocytes and macrophages) taken from the wound periphery in healthy mice and mice with STZ-induced T1DM led to discovery of novel cluster of osteoclast-like macrophages and showed, that the reason for suppressed wound healing in T1DM can be their impaired functioning (124).

During the development of mechanical allodynia (MA), one of the symptoms of diabetic peripheral neuropathy in rats under STZ-induced T1DM, scRNA-seq of neural tissue revealed the transcriptomic changes of somatosensory neurons. Moreover, a novel neuron cluster, MAAC (Mechanical AllodyniaAssociated Cluster), was revealed, and its number was elevated in rats with MA and T1DM. The transcriptomic characteristics of the novel cluster were studied, its origin, transition trajectory, regulons and intercellular interactions were shown. The marker genes upregulated in MAAC were Fxyd7 and Atp1b1, the genes related with NKA sodium-potassium ATPase activity. MAAC was also characterized by high expression of genes related to neurofilaments and cytoskeleton. Atp1b1 expression was most probably driven by the activity of Hobx7 and Larp1 transcription factors. These neurons also tightly interacted with satellite glial cells, but not with other neurons. Taken together, these findings can help to understand the mechanisms of neuropathies in diabetes (125).

Zhong et al. (83) studied immune cells of bone marrow from streptozotocin-induced T1DM mice. The major contributors to progression of osteopenia as T1DM complication were neutrophils and B-lymphocytes of bone marrow. Based on neutrophils-to-lymphocytes ratio that differed between experimental and control samples, quantitative correlations between STZ-induced T1DM and its osteopenic complication were found: the higher N/L ratio, the higher was the risk of diabetic complication (83). Single-cell mRNA sequencing of retinal cells demonstrated that Cirbp, Mt1, Rbm3, Hmgb2, Mt2 are upregulated in mice with diabetic retinopathy in all retinal cells analyzed (rods, cones, Muller¨ cells, retinal glia). T1DM was accompanied by dysregulation of many genes in endothelium and elevation of pro-inflammatory genes in retinal microglia (126). The possible use of these findings can be the application of this gene signature as a prognostic marker for the risk of diabetic retinopathy based on ocular exosome analysis.

Thus, application of scRNA-seq allowed to reveal the pathogenetic mechanisms that facilitate progression of other diabetic complications. This can improve the efficiency of treatment of both diabetes and its complications.

The data mentioned in the review are summarized in **Supplementary Table S1**.

## 7 Conclusions

Modern achievements in the area of single-cell technologies allowed to obtain valuable information on the mechanisms of T1DM pathogenesis, including novel data on the mechanisms of diabetic autoimmunity, such as roles of populations and subpopulations of immune cells, heterogeneity and complexity of islet cell functioning upon T1DM progression or high risks of its onset, and significance of intercellular communications during manifestation or progression of the disease. Novel applications of single-cell technologies in T1DM studies covered by the present review allowed to reveal features of type 1 diabetes mellitus in human and animals that can be analysed in blood cells:

•In T-cells:

 • Transcriptome signatures of CD4^+^ T-cells with high expression of GATA3, CCR4, and IRF4, and low expression of interferon response genes (IFNG, CD69, and GBP5).
 • High expression of IL32 gene in T-lymphocytes and NK-cells in PBMC.

•In B-cells:

 • Overexpression of TCL1A in naive B-cells of peripheral blood.

•In monocytes:

 • High expression of three-gene signature ACTG1, REL, TRIB1, in peripheral monocytes.
 • Enrichment of a cluster of blood and pancreas monocytes with elevated SIGLEC1 expression.

scRNA-seq also helped to reveal biomarkers that could serve as indicators of successful T1DM treatment with teplizumab (IL7R, CD127), and potential targets for monoclonal antibody treatment (TLR4, TRAJ23, IL23). Apart from that, novel markers of T1DM complications were found: genes encoding VCAN, TIMP1, TNC, C3, CP, EIF4B, RICTOR, PRKCB for diabetic kidney disease; Fxyd7 and Atp1b1 for mechanical allodynia; Cirbp, Mt1, Rbm3, Hmgb2, Mt2 for diabetic retinopathy. Thus, scRNA-seq may serve as a method to find a series of T1DM biomarkers that would further be used in diagnostics by a cheaper technique such as reverse transcription real-time PCR or microarray.

In spite of the ability of scRNA-seq to uncover complex interactions between cell populations in such a heterogeneous process as T1DM, several limitations of this method should be mentioned. For the obtained data to be statistically significant, a sufficient number of cells of each subtype is required, while library preparation might be accompanied by losses of the genetic material and low mRNA content in the samples (particularly, when the number of dead cells is high). Second, to avoid the artifacts of scRNA-seq, high quality of the libraries is mandatory. Usually, it is taken into account by filtration and processing of the sequencing data; however, the filtration may reduce the numbers of available cells significantly. Third, scRNA-seq cannot differentiate live and dead cells, and working with fixed biomaterial restricts the application of this method to analyze processes occurring in the pancreas during T1DM. Moreover, it should be noted that analysis of islet cells is a highly invasive procedure, and due to that, most of the studies of islet cells in T1DM have been conducted in animal models. The most relevant studies from the point of view of practical clinical application are those showing T1DM markers in PBMC, but transcription signatures of immune cells in blood are often non-specific and unable to reflect the pathological process of pancreatic cell death.

There are certain limitations of single-cell RNA sequencing compared to other transcriptomic methods. When we compare scRNA-seq and bulk RNA-seq, the filtration of the raw data in scRNA-seq can lead to missing the most minor subpopulations of the transcripts; so, the advantage of assigning a set of transcripts to any cellular subtype is restricted by a number of transcripts required to pass the quality control. Due to that, the information revealed by bulk RNA-sequencing is more complete from the point of view of the amount of data generated, including minor transcripts, but its disadvantage, in turn, is lack of dividing the cells into subtypes. In many cases, the mentioned disadvantage of single-cell RNA-seq can be overcome by generating a combined set of the data – so-called “pseudo-bulk RNA sequencing”.

When compared scRNA-seq with snRNA-seq (single-nucleus RNA sequencing), the last method offers an opportunity to track several transcripts of the genes of interest starting from the polyadenylation event. In other words, snRNA-seq allows to track almost the entire process of mRNA maturation and to catch the intermediate splicing events, which is crucial when studying the influence of splicing on the progression of diseases. In case of single-cell RNA sequencing, the intermediate mRNAs will be masked by a great amount of mature mRNA present in the cytosol.

The events happening in the scRNA-seq studies might be relevant to T1DM studies: apart from currently applied short-read technologies (mostly represented by various Illumina versions), the scientists will address third-generation technologies - nanopore sequencing developed by Oxford Nanopore, and singlemolecule real-time sequencing (SMRT-sequencing) developed by Pacific BioSciences. Oxford Nanopore technology allows to obtain long (several Kb) reads, and it allows to sequence both RNA and DNA, but on a single-cell level, only cDNA is subjected to sequencing. Integration of this technology with 10x Chromium library preparation has been shown (127); another example of combined study by Oxford Nanopore and Illumina is (128). SMRT technology (Pacific BioSciences) has a fidelity rate lower than Illumina yet, but it was recently shown to surpass Oxford Nanopore by fidelity (129). On the whole, we expect that if fidelity of Oxford Nanopore and PacBio sequencing will exceed 99% per read, it will allow their wider application in mRNA sequencing, including single-cell mRNA sequencing, as the sole methods. Such studies are already emerging - for example, there is a study where the fidelity of 99.99% is suggested upon application of demultiplexing in circular consensus sequencing of cDNA library derived from the single-cell transcriptomes (130). However, now the use of ONT and PacBio in tandem with “classical” Illumina or Illumina-like short-read technologies is more relevant, for example, for better mapping of alternative splicing variants that cannot always be mapped correctly using only short-read Illumina technology.

In summary, we should emphasize that single-cell RNA sequencing is a modern and actively developing and promising method for examination of numerous pathologies and elaborating the approaches for their treatment. Particularly, in type 1 diabetes mellitus studies, this method is applied for both pathogenetic studies revealing the details of disease progression mechanisms and for more applied studies such as determination of methods for avoiding islet graft rejection, monitoring the efficiency of the treatment, prognostics and early diagnostics of T1DM. In the nearest future, we should expect a significant increase in the number of studies on applying different variations of scRNA-seq in studying this disease.
