# Supplementary material for: Single-cell RNA sequencing in studies of type 1 diabetes mellitus: modern state-of-the-art and technical peculiarities
Source: Front Endocrinol (Lausanne). 2025 Sep 5;16:1663728. doi: 10.3389/fendo.2025.1663728 (PMC12446015; doi:10.3389/fendo.2025.1663728)
Supplement: Supplementary file 1 [file DataSheet1.pdf]

Supplementary Material

Table S1: Studies on single-cell RNA sequencing applied to T1DM studies. Objects, main results and references are listed.

Tissue	Organism	Authors, year	Cell type	Reference groups	Main results
Blood	Human	Okamura, 2022*	CD8+ T-cells, FOXP3+ T-cells	T1DM	T1DM leads to significant upregulation of cytotoxicity gene expression in CD8+ -cells and to increased expression of IL-4 and TNFRSF4 in Tregs, showing deficiency of phenotype and suppression capacity of Tregs (Okamura et al. (2022))
Blood	Human	Honardoost, 2024	PBMC (T-cells, B-cells, NK-cells, monocytes)	T1DM stage 3 / healthy	A z-score of T1DM-related transcriptomic changes was proposed (TMZ), it correlates with presence of immune markers of T1DM and with response to pharmacological treatment of T1DM in clinical trials (Honardoost et al. (2024))

Table S1 –

Tissue	Organism	Authors, year	Cell type	Reference groups	Main results
Blood	Human	Eugster et al, 2024	PBMC	T1DM first onset/healthy	GAD65-specific responses are characteristic for many T-cell phenotypes in healthy persons and in T1DM. Occurrence of GADA is conditioned physiologically and related to intense neonatal pancreas remodeling. Convergent GADA clonotypes in central memory cells subpopulations are expanded in T1DM patients (Eugster et al. (2024))
Blood	Human	Okamura 2025	PBMC	T1DM / healthy	Upon stimulation of PBMC from patients with T1DM by overlapping antigen peptides of GAD, IA-2 and insulin, higher autoimmune response was observed after insulin action, and it was associated with TCR combinations TRB TRBV28/TRBJ2-7 and TRA TRAV12-2/TRAJ18 on cytotoxic T-cells (Okamura et al. (2025))

Table S1 –

Tissue	Organism	Authors, year	Cell type	Reference groups	Main results
Pancreas	Human	Wang et al., 2021	β-cells and β-like cells	T1DM / healthy	A correlation of rs3842753 C/C genotype with innate elevated insulin gene and EPR-dependent stress markers expression was revealed, reflecting predisposition to T1DM (Wang et al. (2021))
Pancreas	Human	Bosi et al., 2022	α-cells of islets	T1DM / T2DM	Transcription profile differences of α-cells in T1DM and T2DM was revealed: T1DM demonstrated elevated expression of genes involved into autoimmune processes (Bosi et al. (2022))
Pancreas	Human	Muños-Garcia et al., 2024	Islet and duct cells	T1DM / healthy / Wolfrahm syndrome	A leading role of duct cells in enhancement of inflammatory response was shown in T1DM (Muñoz García et al. (2024))
Pancreas	Human	Patil et al., 2024	islet cells	T1DM stage 3 / T1DM stage 2 / healthy	Machine-learning methods on base of scRNA-seq data allow to predict the risk of T1DM progression by gene signatures (Patil et al. (2024))

Table S1 –

**Tissue**	**Organism**	**Authors, year**	**Cell type**	**Reference groups**	**Main results**
Pancreas	Human and mouse	Kang et al., 2023**	islet cells (human donor and islet grafted into mouse)	healthy (human) / immune deficiency (mouse)	A variant of clusterization of islet cells was proposed and transcriptional changes in different islet cell subtypes upon their transplantation were revealed (Kang et al. (2023))
Pancreas	Mouse	Chen et al., 2022b**	Grafted islet cells: immune and endocrine	T1DM after allogeneic islet transplantation	The main immune cells in allogeneic grafts are T-cells and myeloid cells, while islet cells begin to resemble antigen-presenting cells (**?**)
Pancreas	Mouse	Zhou et al., 2024**	Grafted islet cells: immune and endocrine	Syngeneic grafts / allogeneic grafts	In different graft models, subpopulations of T-cells are activated via different pathways, showing their specific roles in immune graft rejection (Zhou et al. (2024))

Table S1 –

Tissue	Organism	Authors, year	Cell type	Reference groups	Main results
Pancreas	Mouse	Feng et al., 2020	Islet and duct cells, immune cells	T1DM	In normal and pathological conditions, there are several subpopulations of pancreatic β-cells with different functional abilities, sensitivity to diabetogenic factors and transcription profiles, which is determined by different GLUT2 expression level. After the death of highly functional β-cells during the course of T1DM, they cannot be replenished by dedifferentiation and redifferentiation neither from low-functinality β-cells with low GLUT2, level, nor from α-cells (Feng et al. (2020))
Pancreas	Mouse	Zakharov, 2020	Immune cells, endothelial cells, mesenchymal cells	T1DM, early, middle and late stages (4, 8 and 15 weeks)	Heterogeneity of transcription patterns at different stages of diabetes was shown, reflecting the complex nature of the pathological process and different roles of immune cells at different stages of the disease (Zakharov et al. (2020))

Table S1 –

**Tissue**	**Organism**	**Authors, year**	**Cell type**	**Reference groups**	**Main results**
Pancreas	Mouse	Stancill, 2021	Islet cells treated with IL-1β, IFN-γ	healthy	All the endocrine cells of pancreas demonstrated transcriptional responses to cytokines, particularly, to IL-1β (Stancill et al. (2021))
Pancreas	Mouse	Ji, Guo, 2023	Islet cells: immune and endocrine	T1DM, early, middle and late stages (4, 8 and 15 weeks)	Suppression of NKG7 prevents T-cell proliferations; suppression of C1QB blocks differentiation of monocytes into macrophages. Both processes alleviate the autoimmune islet degradation (Ji and Guo (2023))
Kidney	Human	Lu, 2022	Immune cells	T1DM / T1DM + nephropathy/ healthy	Immune cells express mTOR signaling pathway genes at a high level, it is one of the key mechanisms of diabetic nephropathy progression (Lu et al. (2022))
Bone marrow	Mouse Rat	Zhong, 2022	Neutrophils and B-cells	T1DM / healthy	Quantitative and qualitative changes in neutrophils and B-cells were revealed in T1DM group (249 DEGs). A correlation of the changes with osteopenia was shown (Zhong et al. (2022))

Table S1 –

Tissue	Organism	Authors, year	Cell type	Reference groups	Main results
Ganglion	Mouse	Zhou et al., 2022	Neurons and glial cells	T1DM / T1DM + neuropathy/ healthy	A novel cluster of neurons associated with mechanical allodynia, MAAC, was identified. Its role in diabetic neuropathy progression was shown, and its origin was suggested to be PEP line (peptidergic nociceptors) (Zhou et al. (2022))
Skin	Mouse	Ma, 2023	CD45+ cells (T-cells, neutrophils, macrophages, monocytes)	T1DM/healthy control	Impairment of osteoclast-like macrophage function in T1DM can be the cause of defective wound healing in this disease (Ma et al. (2023))
Retina	Mouse	Sun, 2021	All retinal cells	T1DM + retinopathy / control	T1DM leads to impairment of regulation of multiple genes activity in retinal endothelion and to overexpression of pro-inflammatory cytokines in retinal microglia (Sun et al. (2021))

REFERENCES

Bosi, E., Marchetti, P., Rutter, G. A., and Eizirik, D. L. (2022). enHuman alpha cell transcriptomic signatures of types 1 and 2 diabetes highlight disease-specific dysfunction pathways. *iScience* 25, 105056. doi:10.1016/j.isci.2022.105056

Eugster, A., Lorenc, A., Kotrulev, M., Kamra, Y., Goel, M., Steinberg-Bains, K., et al. (2024). enPhysiological and pathogenic T cell autoreactivity converge in type 1 diabetes. *Nature Communications* 15, 9204. doi:10.1038/s41467-024-53255-9

Feng, Y., Qiu, W.-L., Yu, X.-X., Zhang, Y., He, M.-Y., Li, L.-C., et al. (2020). enCharacterizing pancreatic - cell heterogeneity in the streptozotocin model by single-cell transcriptomic analysis. *Molecular Metabolism*

37, 100982. doi:10.1016/j.molmet.2020.100982

Honardoost, M. A., Adinatha, A., Schmidt, F., Ranjan, B., Ghaeidamini, M., Arul Rayan, N., et al. (2024). enSystematic immune cell dysregulation and molecular subtypes revealed by single-cell RNA-seq of subjects with type 1 diabetes. *Genome Medicine* 16, 45. doi:10.1186/s13073-024-01300-z

Ji, L. and Guo, W. (2023). enSingle-cell RNA sequencing highlights the roles of C1QB and NKG7 in the pancreatic islet immune microenvironment in type 1 diabetes mellitus. *Pharmacological Research* 187, 106588. doi:10.1016/j.phrs.2022.106588

[Dataset] Kang, R. B., Lee, J., Varela, M., Li, Y., Rosselot, C., Zhang, T., et al. (2023). enHuman Pancreatic -Cell Heterogeneity and Trajectory Inference Analysis Using Integrated Single Cell- and Single Nucleus-RNA Sequencing Platforms. doi:10.1101/2023.11.19.567715

Lu, X., Li, L., Suo, L., Huang, P., Wang, H., Han, S., et al. (2022). Single-Cell RNA Sequencing Profiles Identify Important Pathophysiologic Factors in the Progression of Diabetic Nephropathy. *Frontiers in Cell and Developmental Biology* 10, 798316. doi:10.3389/fcell.2022.798316

Ma, J., Song, R., Liu, C., Cao, G., Zhang, G., Wu, Z., et al. (2023). enSingle-cell RNA-Seq analysis of diabetic wound macrophages in STZ-induced mice. *Journal of Cell Communication and Signaling* 17, 103–120. doi:10.1007/s12079-022-00707-w

Muñoz García, A., Juksar, J., Groen, N., Zaldumbide, A., De Koning, E., and Carlotti, F. (2024). Single-cell transcriptomics reveals a role for pancreatic duct cells as potential mediators of inflammation in diabetes mellitus. *Frontiers in Immunology* 15, 1381319. doi:10.3389/fimmu.2024.1381319

Okamura, T., Hamaguchi, M., Tominaga, H., Kitagawa, N., Hashimoto, Y., Majima, S., et al. (2022). enCharacterization of Peripheral Blood TCR in Patients with Type 1 Diabetes Mellitus by BD RhapsodyTM VDJ CDR3 Assay. *Cells* 11, 1623. doi:10.3390/cells11101623

Okamura, T., Kitagawa, N., Kitagawa, N., Sakai, K., Sumi, M., Kobayashi, G., et al. (2025). enSingle-cell analysis reveals islet autoantigen's immune activation in type 1 diabetes patients. *Journal of Clinical Biochemistry and Nutrition* 76, 64–84. doi:10.3164/jcbn.24-86

Patil, A. R., Schug, J., Liu, C., Lahori, D., Descamps, H. C., Naji, A., et al. (2024). enModeling type 1 diabetes progression using machine learning and single-cell transcriptomic measurements in human islets. *Cell Reports Medicine* 5, 101535. doi:10.1016/j.xcrm.2024.101535

Stancill, J. S., Kasmani, M. Y., Khatun, A., Cui, W., and Corbett, J. A. (2021). enSingle-cell RNA sequencing of mouse islets exposed to proinflammatory cytokines. *Life Science Alliance* 4, e202000949. doi:10.26508/lsa.202000949

Sun, L., Wang, R., Hu, G., Liu, H., Lv, K., Duan, Y., et al. (2021). enSingle cell RNA sequencing (scRNA-Seq) deciphering pathological alterations in streptozotocin-induced diabetic retinas. *Experimental Eye Research* 210, 108718. doi:10.1016/j.exer.2021.108718

Wang, S., Flibotte, S., Camunas-Soler, J., MacDonald, P. E., and Johnson, J. D. (2021). enA New Hypothesis for Type 1 Diabetes Risk: The At-Risk Allele at rs3842753 Associates With Increased Beta-Cell INS Messenger RNA in a Meta-Analysis of Single-Cell RNA-Sequencing Data. *Canadian Journal of Diabetes* 45, 775–784.e2. doi:10.1016/j.jcjd.2021.03.007

Zakharov, P. N., Hu, H., Wan, X., and Unanue, E. R. (2020). enSingle-cell RNA sequencing of murine islets shows high cellular complexity at all stages of autoimmune diabetes. *Journal of Experimental Medicine* 217, e20192362. doi:10.1084/jem.20192362

Zhong, J., Mao, X., Li, H., Shen, G., Cao, X., He, N., et al. (2022). enSingle-cell RNA sequencing analysis reveals the relationship of bone marrow and osteopenia in STZ-induced type 1 diabetic mice. *Journal of Advanced Research* 41, 145–158. doi:10.1016/j.jare.2022.01.006

Zhou, H., Pu, Z., Lu, Y., Zheng, P., Yu, H., and Mou, L. (2024). Elucidating T cell dynamics and molecular mechanisms in syngeneic and allogeneic islet transplantation through single-cell RNA sequencing. *Frontiers in Immunology* 15, 1429205. doi:10.3389/fimmu.2024.1429205

Zhou, H., Yang, X., Liao, C., Chen, H., Wu, Y., Xie, B., et al. (2022). The Development of Mechanical Allodynia in Diabetic Rats Revealed by Single-Cell RNA-Seq. *Frontiers in Molecular Neuroscience* 15, 856299. doi:10.3389/fnmol.2022.856299
